# Hepatocellular heme oxygenase 1 deficiency does not affect inflammatory hepcidin regulation in mice

**DOI:** 10.1371/journal.pone.0219835

**Published:** 2019-07-11

**Authors:** Edouard Charlebois, Carine Fillebeen, Kostas Pantopoulos

**Affiliations:** Lady Davis Institute for Medical Research, Jewish General Hospital, and Department of Medicine, McGill University, Montreal, Quebec, Canada; Medizinische Fakultat der RWTH Aachen, GERMANY

## Abstract

Hepcidin is an iron regulatory peptide hormone that is secreted from hepatocytes and inhibits iron efflux from tissues to plasma. Under inflammatory conditions, hepcidin is transcriptionally induced by IL-6/STAT3 signaling and promotes hypoferremia, an innate immune response to infection. If this pathway remains unresolved, chronic overexpression of hepcidin contributes to the anemia of inflammation, a common medical condition. Previous work showed that carbon monoxide (CO) releasing drugs (CORMs) can attenuate inflammatory induction of hepcidin. Because CO is physiologically generated during heme degradation by heme oxygenase 1 (HO-1), an IL-6-inducible enzyme with anti-inflammatory properties, we hypothesized that hepatocellular HO-1 may operate as a physiological feedback regulator of hepcidin that resolves inflammatory signaling. To address this, we generated and analyzed hepatocyte-specific HO-1 knockout (Hmox1^Alb-Cre^) mice. We show that these animals mount appropriate hepcidin-mediated hypoferremic response to LPS-induced inflammation, with kinetics similar to those of control Hmox1^fl/fl^ mice. Likewise, primary hepatocytes from Hmox1^Alb-Cre^ and Hmox1^fl/fl^ mice exhibit similar degree and kinetics of hepcidin induction following IL-6 treatment. We conclude that hepatocellular HO-1 has no physiological function on hepcidin regulation by the inflammatory pathway.

## Introduction

Heme oxygenase 1 (HO-1) is a stress-inducible microsomal enzyme that catalyzes the oxidative degradation of heme to ferrous (Fe^2+^) iron, biliverdin and carbon monoxide (CO). It is highly expressed in macrophages and plays a key role in recycling of heme iron from senescent red blood cells during erythrophagocytosis [1, 2]. HO-1 is widely viewed as a cytoprotective and anti-inflammatory enzyme by clearing cytotoxic and pro-inflammatory free heme, but also by promoting immunoregulatory responses via its reaction products biliverdin/bilirubin and CO. Beneficial effects of HO-1 induction in various models of cell injury can be mimicked by CO-releasing molecules (CORMs), which in many instances recapitulate physiological signaling functions of CO [3].

The CO-releasing drugs CORM-2 and CORM-3 were shown to inhibit induction of hepcidin in response to acute inflammation or endoplasmic reticulum (ER) stress in human hepatoma cells (HepG2 and Huh7) and in mice [4]. Hepcidin is a peptide hormone that controls systemic iron homeostasis [5]. It is secreted from hepatocytes and inactivates the iron exporter ferroportin in macrophages, enterocytes and other cell types, preventing iron efflux to the bloodstream. The *Hamp* gene encoding hepcidin is transcriptionally induced in response to iron, inflammation or ER stress. Inflammatory induction of hepcidin involves IL-6/STAT3 signaling and is considered to be protective during infection as an innate immune response that deprives bacteria from iron, an essential nutrient. Resolution of this response is critical to prevent persistent hypoferremia, a major contributor to the anemia of inflammation [6]. This is a frequent complication in patients suffering from chronic inflammatory disorders and constitutes the most prevalent anemia in industrial countries, and the second most prevalent anemia worldwide.

Considering that HO-1 is likewise induced by the IL-6/STAT3 pathway in hepatocytes [7], and based on the pharmacological data on hepcidin regulation by CO-releasing drugs [4], we hypothesized that hepatocellular HO-1 may physiologically serve to attenuate inflammatory induction of hepcidin. We addressed this hypothesis using mice with hepatocyte-specific HO-1 ablation.

## Materials and methods

### Animals

Homozygous Hmox1^fl/fl^:Alb-Cre mice were generated by breeding Hmox1^fl/fl^ (kindly provided by Dr. Kollias, BSRC Al. Fleming, Greece) [8] with Alb-Cre mice [9] (B6.Cg-Tg(Alb-cre)21Mgn/J; purchased from Charles River Laboratories, Cambridge, MA). Genotyping was performed with gene-specific primers [10]. All mice were housed according to institutional guidelines [11]; they were given free access to water and a standard rodent diet. Where indicated, mice were injected intraperitoneally with 38.44 μmol/kg body weight heme arginate (Normosang; Leiras Oy, Finland) diluted in phosphate-buffered saline, or 1 μg/g body weight lipopolysaccharide (LPS; Sigma-Aldrich, L2280 O55:B5, purified by phenol extraction). Control mice were injected with phosphate-buffered saline. At the endpoint, animals were sacrificed by CO_2_ inhalation followed by cervical dislocation. Experimental procedures were approved by the Animal Care Committee of McGill University (protocol 4966).

### Primary murine hepatocytes

Primary hepatocytes were isolated from mouse livers by using a modified two-step collagenase perfusion method [11]. The cells were treated with 20 ng/ml murine IL-6 (Cell Signaling).

### Serum biochemistry

Serum iron and total iron binding capacity (TIBC) were measured on a Roche Hitachi 917 Chemistry Analyzer at the Biochemistry Department of the Jewish General Hospital. Transferrin saturation was calculated from the ratio of serum iron and TIBC.

### Biochemical assays and histology

Quantitative real-time (qPCR), Western blotting and immunohistochemistry were performed as described previously [10–12].

### Liver Iron Content

Liver nonheme iron was quantified using the ferrozine assay as described previously [13].

### Statistical analysis

Statistical analysis was performed by using the Prism GraphPad software (version 7.0e). Multiple groups were subjected to analysis of variance (ANOVA) with Bonferroni post-test comparison. A probability value p<0.05 was considered statistically significant.

## Results and discussion

**Fig 1:**
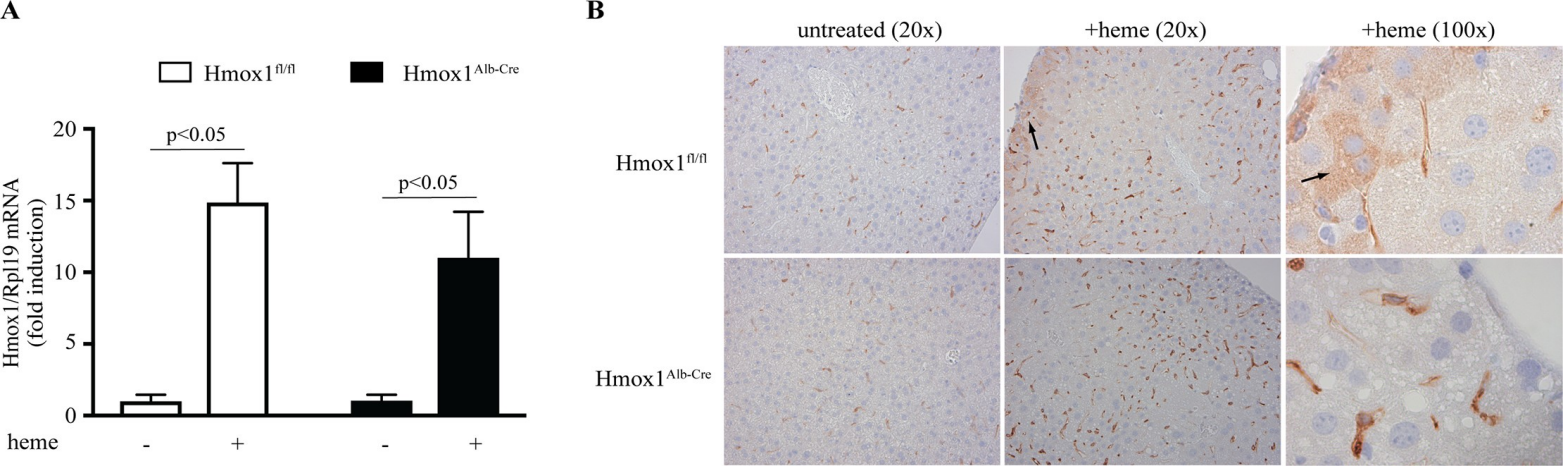
Hmox1^Alb-Cre^ mice bear hepatocyte-specific disruption of HO-1. 8-week old male Hmox1^Alb-Cre^ mice and Hmox1^fl/fl^ littermates (n = 5–7 for each experimental group) were left untreated or injected with 38.44 μmol/kg body weight heme arginate. After 18 hours the animals were sacrificed, and livers were processed for preparation of RNA or fixed for immunohistochemistry. (A) qPCR analysis of liver *Hmox1* mRNA. (B) Immunohistochemical detection of HO-1 in liver sections (magnification: 20x and 100x); arrows indicate hepatocellular HO-1. Data in (A) are presented as the mean ± SEM. Statistical analysis was performed by one-way ANOVA.

Hmox1^fl/fl^:Alb-Cre (referred to as Hmox1^Alb-Cre^) mice contain floxed *Hmox1* alleles and express the Cre recombinase under control of the hepatocyte-specific *Alb* (albumin) promoter, which is activated in fetal and neonatal mouse liver upon hepatocyte differentiation [14]. Thus, the Hmox1^Alb-Cre^ mice are expected to lack HO-1 expression in hepatocytes. To validate this, we analyzed *Hmox1* (HO-1) mRNA from livers of untreated Hmox1^fl/fl^ and Hmox1^Alb-Cre^ mice, or from animals previously injected with the HO-1 inducer heme arginate. The data in Fig 1A show a robust upregulation of “whole liver” *Hmox1* mRNA after 18 hours of heme arginate injection in both genotypes. Presumably, this is due to high abundance of HO-1 in Kupffer cells, the major HO-1-expressing liver cells [15], which skews differences in hepatocellular Hmox1 mRNA content. Immunohistochemical analysis of liver sections confirmed this notion since the strong HO-1 signal is retained in Kupffer cells from both genotypes (Fig 1B). However, HO-1 expression was only evident in hepatocytes from heme arginate-treated Hmox1^fl/fl^ mice (arrows) but not Hmox1^Alb-Cre^ mice. We further corroborated these data *in vitro* using isolated primary murine hepatocytes. Following treatment with heme arginate, time-dependent *Hmox1* mRNA induction was only observed in control Hmox1^fl/fl^ but not Hmox1^Alb-Cre^ hepatocytes (S1 Fig). Notably, heme arginate triggered a suppression of *Hamp* (hepcidin) mRNA; this response was quantitatively indistinguishable among the genotypes and consistent with the known hepcidin inhibition by inorganic iron *in vitro* [16]. The above data suggest efficient hepatocellular HO-1 disruption in Hmox1^Alb-Cre^ mice and are in line with previous relevant findings [17].

**Fig 2:**
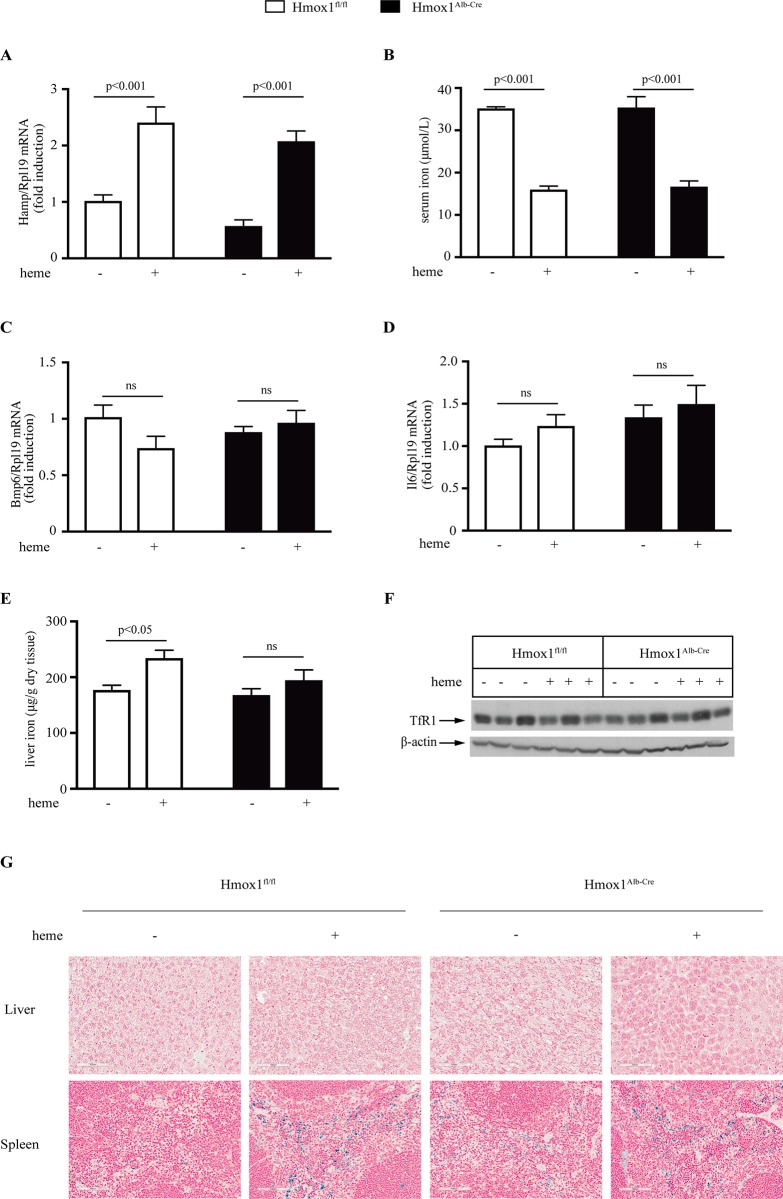
The heme arginate treatment does not promote systemic iron overload. Sera from mice described in Fig 1 were collected for iron biochemistry and livers were used for iron quantification, preparation of protein extracts, or preparation of RNA; liver and splenic sections were used for histology. (A) qPCR analysis of *Hamp* mRNA. (B) Serum iron. (C) qPCR analysis of *Bmp6* mRNA. (D) qPCR analysis of *Il6* mRNA. (E) Liver iron quantification by the ferrozine assay. (F) Western blot analysis of TfR1 and β-actin (arrows). (G) Perls staining of liver and spleen sections (magnification 20x). All data are presented as the mean ± SEM. Statistical analysis was performed by two-way ANOVA.

We further characterized the mice treated with heme arginate for 18 h and observed a robust ~2-fold *Hamp* mRNA induction in the liver, accompanied by an expected drop in serum iron in both genotypes (Fig 2A and 2B). The induction of hepcidin was not accompanied by increased expression of *Bmp6* or *Il6* (IL-6) mRNAs (Fig 2C and 2D), which are markers of iron or inflammatory signaling, respectively. Nevertheless, it is conceivable that iron and/or inflammatory signaling pathways were activated earlier and returned to baseline at the endpoint. Considering that ubiquitous Hmox1-/- mice develop progressive iron overload [18], we measured liver nonheme iron levels but found no differences among the genotypes (Fig 2E). Interestingly, a small but significant increase in liver iron was noted in Hmox1^fl/fl^ but not the Hmox1^Alb-Cre^ mice after heme arginate injection. Nevertheless, expression of transferrin receptor 1 (TfR1), a marker of iron load, did not appear to be significantly affected by the treatment (Fig 2F). Along these lines, staining of liver sections with Perls Prussian Blue did not reveal non-heme iron deposits, irrespectively of treatment or genotype (Fig 2G, top). However, the staining appeared more intense in splenic macrophages of both genotypes following heme arginate treatment (Fig 2G, bottom). These data suggest that under the above experimental conditions, the heme arginate treatment does not promote substantial systemic iron overload. The intense staining for non-heme iron in splenic sections is consistent with heme catabolism in macrophages.

**Fig 3:**
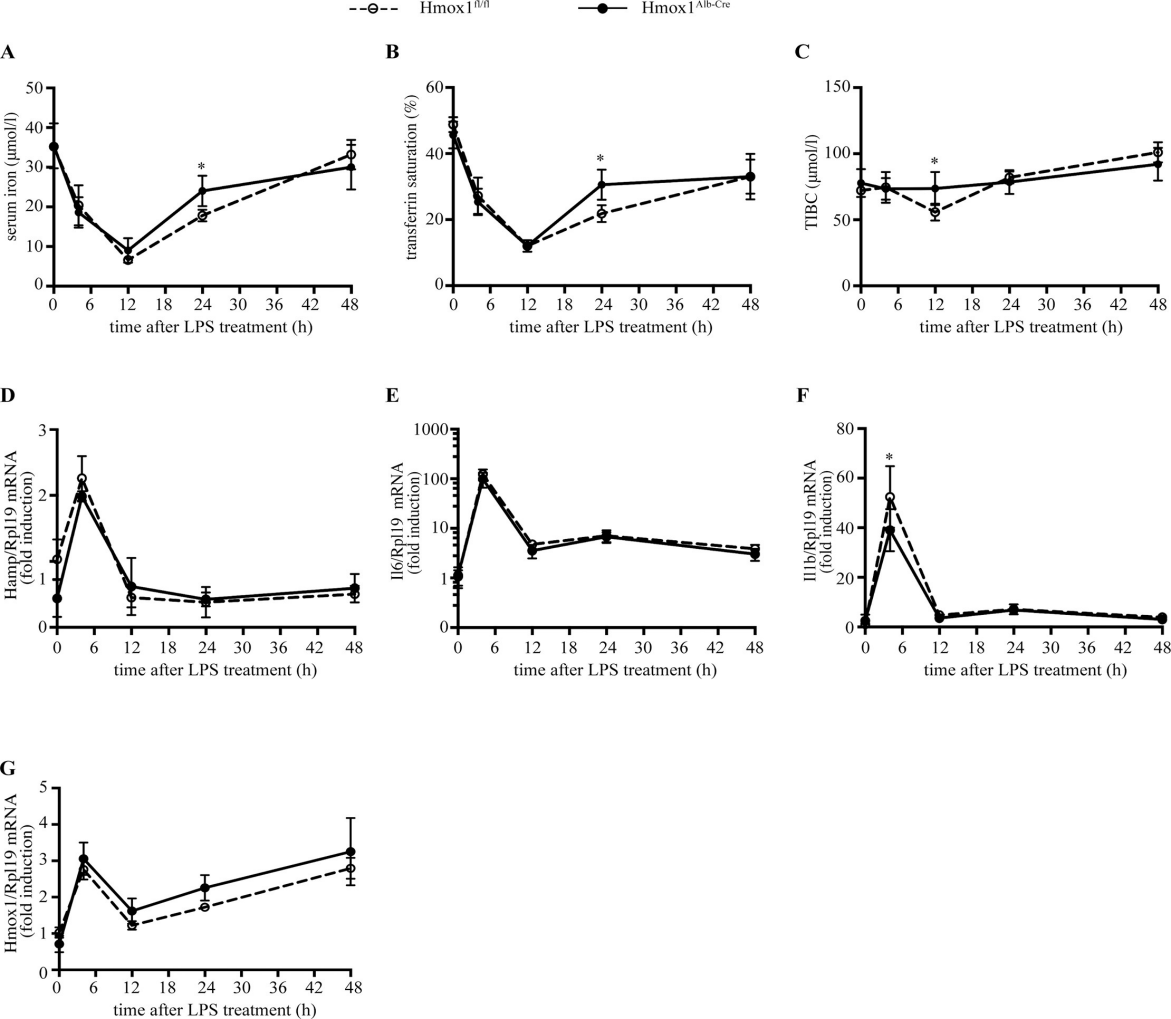
Hmox1^Alb-Cre^ mice develop appropriate hypoferremia and exhibit physiological hepcidin regulation in response to LPS-induced acute inflammation. 8-week old male Hmox1^Alb-Cre^ mice and Hmox1^fl/fl^ littermates (n = 5–6 for each experimental group) were injected with phosphate-buffered saline or injected with 1 μg/g body weight LPS for the indicated time intervals. At the endpoints the animals were sacrificed; sera were collected and used for iron biochemistry, and livers were processed for preparation of RNA. (A) Serum iron. (B) Transferrin saturation. (C) Total iron binding capacity (TIBC). (D) qPCR analysis of liver *Hamp* mRNA. (E) qPCR analysis of liver *Il6* mRNA. (F) qPCR analysis of liver *Il1b* mRNA. (G) qPCR analysis of *Hmox1* mRNA. All data are presented as the mean ± SEM. Statistical analysis was performed by two-way ANOVA. Statistically significant differences across genotypes are indicated by * (p<0.05).

**Fig 4:**
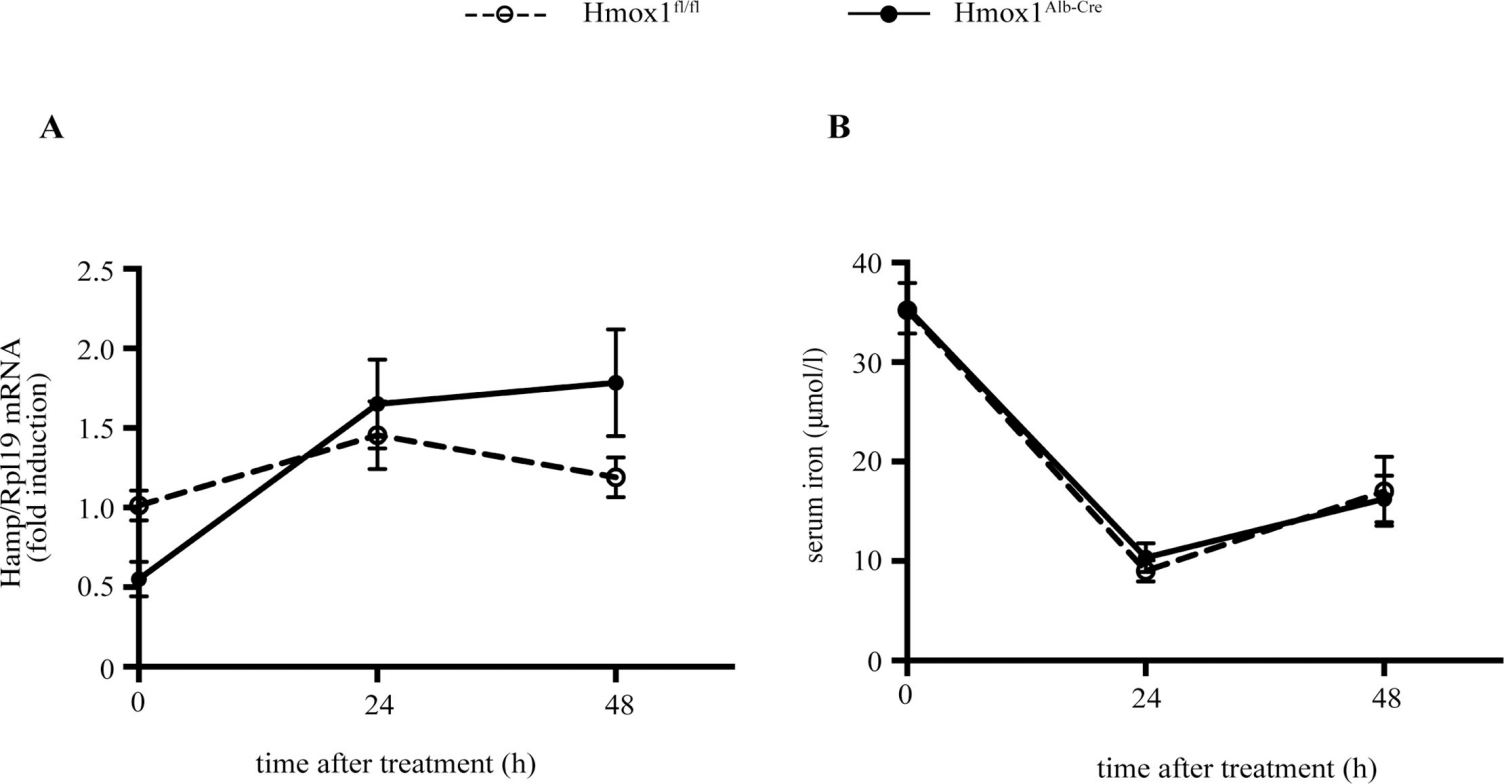
Hmox1^Alb-Cre^ and Hmox1^fl/fl^ mice exhibit similar responses to combined challenge with heme arginate and LPS. 8-week old male Hmox1^Alb-Cre^ mice and Hmox1^fl/fl^ littermates (n = 4–5 for each experimental group) were injected with phosphate-buffered saline or injected with both 38.44 μmol/kg body weight heme arginate and 1 μg/g body weight LPS for the indicated time intervals. At the endpoints the animals were sacrificed; sera were collected and used for iron biochemistry, and livers were processed for preparation of RNA. (A) qPCR analysis of *Hamp* mRNA. (B) Serum iron. All data are presented as the mean ± SEM. Statistical analysis was performed by two-way ANOVA.

We then examined whether the lack of hepatocellular HO-1 affects the recovery of Hmox1^Alb-Cre^ mice from hepcidin-mediated hypoferremic response to inflammation. To this end, Hmox1^Alb-Cre^ and control Hmox1^fl/fl^ littermates were injected with LPS. The animals were euthanized at timepoints ranging from 0–48 hours, and sera and livers were procured for analysis. As expected [11], LPS triggered an ~80% drop in serum iron and transferrin saturation, which peaked at 12 h and was preceded by a ~2-fold induction of *Hamp* (hepcidin) mRNA, a ~100-fold induction of *Il6* (IL-6) mRNA and a ~50-fold induction of Il1b (IL-1β) mRNA at 4 h (Fig 3). Notably, there was no significant difference among the genotypes in the severity of the hypoferremic response, or in the recovery kinetics (with the exception of the 24 h time point, where the recovery of Hmox1^Alb-Cre^ mice appeared more pronounced). Likewise, the degree in “whole liver” *Hamp*, *Il6*, *Il1b* and *Hmox1* mRNA induction, as well as the respective kinetics were similar in Hmox1^Alb-Cre^ and Hmox1^fl/fl^ mice. Considering that heme is a proinflammatory alarmin [19], in another experiment, mice were injected with both heme arginate and LPS and analyzed after 0, 24 or 48 h. There was no significant difference among the genotypes in the kinetics of Hamp mRNA expression (Fig 4A) or recovery from hypoferremia (Fig 4B). Taken together, the data in Figs 3 and 4 indicate that hepatocellular HO-1 does not affect inflammatory hepcidin regulation and the course of the ensuing hypoferremia in mice.

**Fig 5:**
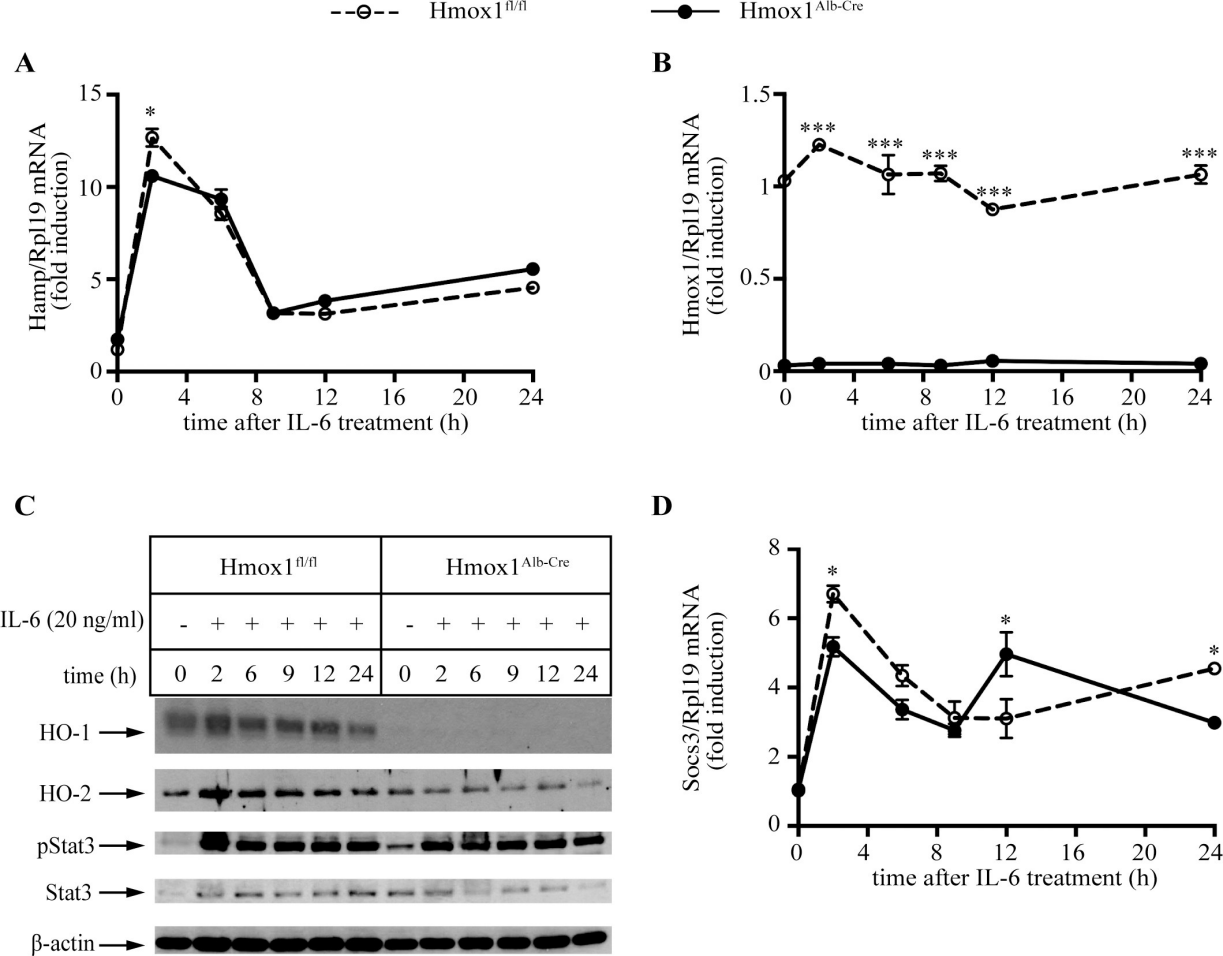
HO-1 deficiency does not affect IL-6-mediated signaling to hepcidin in primary murine hepatocytes. Primary hepatocytes were isolated from livers of Hmox1^Alb-Cre^ and Hmox1^fl/fl^ mice. The cells were cultured in serum-free media and treated with 20 ng/ml murine IL-6. The incubation was terminated at the indicated time intervals and the cells were harvested and lysed. Hepatocyte RNA and protein extracts were analyzed by qPCR and Western blotting, respectively. (A) qPCR analysis of *Hamp* mRNA. (B) qPCR analysis of *Hmox1* mRNA. (C) Western blot analysis of HO-1, HO-2, phospho-Stat3, Stat3 and β-actin (arrows). (D) qPCR analysis of *Socs3* mRNA. Data in graphs are presented as the mean ± SEM. Statistical analysis was performed by two-way ANOVA. Statistically significant differences across genotypes are indicated by * (p<0.05) or *** (p<0.001).

We further addressed whether the lack of HO-1 interferes with inflammatory hepcidin regulation using primary hepatocytes from Hmox1^Alb-Cre^ and Hmox1^fl/fl^ mice. Treatment of the cells with IL-6 promoted a similar ~12.5-fold induction of *Hamp* mRNA in both genotypes, which peaked at 2 h; values returned to baseline after 9 h (Fig 5A). Thus, these data validate the lack of hepatocellular HO-1 involvement in inflammatory hepcidin regulation. Analysis of *Hmox1* mRNA by qPCR (Fig 5B) and HO-1 by Western (Fig 5C) confirmed the efficient HO-1 ablation in hepatocytes from Hmox1^Alb-Cre^ mice. To exclude the possibility of a compensatory response by HO-2, the constitutive HO-1 homologue [20], we determined HO-2 protein levels; as expected, there was no HO-2 induction in Hmox1^Alb-Cre^ hepatocytes. The modest induction of *Hmox1* mRNA and HO-1 in IL-6-treated Hmox1^fl/fl^ hepatocytes may be related to the relatively low dose of IL-6 that was used. Another reason could be that basal *Hmox1* mRNA and HO-1 expression were already high under our experimental conditions (see Fig 5B and 5C), possibly due to stress induction during hepatocyte preparation.

The IL-6 treatment elicited Stat3 phosphorylation (Fig 5C) and stimulated expression of *Socs3* mRNA (Fig 5D), which encodes a feedback inhibitor of the Jak/Stat signaling pathway and target of CO-releasing drugs [4], in both Hmox1^Alb-Cre^ and Hmox1^fl/fl^ hepatocytes. Interestingly, high levels of phosphorylated Stat3 were sustained past 9 h and up to 24 h, in a time frame where *Hamp* mRNA expression was normalized. Similar results were obtained in an *in vivo* time course experiment with LPS-treated wild type mice [11] and may suggest that prolonged Stat3 signaling cannot prevent resolution of the inflammatory hepcidin response. We also noted that basal levels of pStat3 were elevated in Hmox1^Alb-Cre^ hepatocytes. To explore whether this trend is persistent, we analyzed basal Stat3 phosphorylation in biological replicates of untreated Hmox1^Alb-Cre^ and Hmox1^fl/fl^ hepatocytes (n = 5 for each genotype). In spite of an apparent variability, these data reproduce the tendency of higher pStat3 levels in untreated Hmox1^Alb-Cre^ hepatocytes (S2 Fig). We speculate that this may be related to oxidative stress, a known inducer of Stat3 phosphorylation [21], due to lack of antioxidant HO-1.

We conclude that hepatocellular HO-1 does not suffice to mimic the reported pharmacological modulation of hepcidin by CO-releasing drugs [4]. This is consistent with earlier data obtained with cultured HepG2 and Hep3B hepatoma cells, where pharmacological HO-1 induction or inhibition did not affect Hamp mRNA response to IL-6 treatment for 6 h [22]. It is conceivable that HO-1 expressed in hepatocytes may fail to produce sufficient levels of CO to impinge on hepcidin expression due to low abundance. Nevertheless, in another setting, induction of hepatocellular HO-1 recapitulated protective effects of CO-releasing drugs against ethanol-induced hepatotoxicity [23]. Inasmuch as CO is a diffusible signaling molecule [3], our data do not exclude the possibility for a physiological role of HO-1 present in Kupffer cells as a hepcidin regulator in neighboring hepatocytes, but this awaits experimental validation.

## Supporting information

S1 Fig Effects of heme arginate on *Hmox1* and *Hamp* expression in primary murine hepatocytes. Primary hepatocytes were isolated from livers of Hmox1^Alb-Cre^ and Hmox1^fl/fl^ mice. The cells were cultured in serum-free media and treated with 38.44 μM heme arginate. The incubation was terminated at the indicated time intervals and the cells were harvested and used for RNA preparation. (A) qPCR analysis of *Hmox1* mRNA. (B) qPCR analysis of *Hamp* mRNA. Data in graphs are presented as the mean ± SEM. Statistical analysis was performed by two-way ANOVA. Statistically significant differences across genotypes are indicated by *** (p<0.001).
(TIF)

S2 Fig HO-1 deficiency is associated with a tendency for increased Stat3 phosphorylation in primary murine hepatocytes. Primary hepatocytes were isolated from livers of Hmox1^Alb-Cre^ and Hmox1^fl/fl^ mice. The cells were cultured in serum-free media and then harvested and used for preparation of protein lysates. (A) Western blot analysis of pStat3, Stat3 and β-actin (arrows).
(TIF)
