# Supplementary figures and images for: Hepatocellular heme oxygenase 1 deficiency does not affect inflammatory hepcidin regulation in mice
Source: PLoS One. 2019 Jul 11;14(7):e0219835. doi: 10.1371/journal.pone.0219835 (PMC6623421; doi:10.1371/journal.pone.0219835)

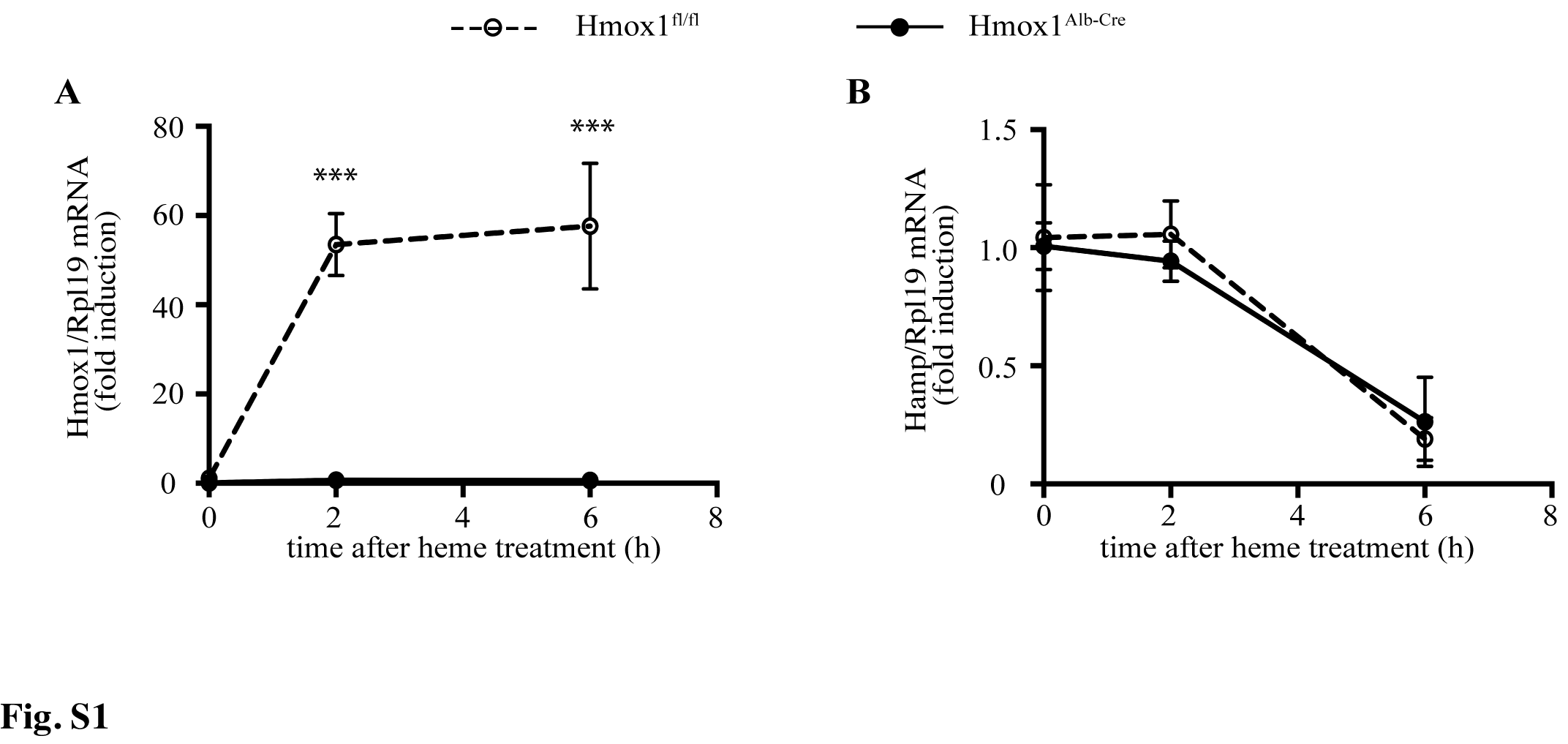

Supplement: S1 Fig — Primary hepatocytes were isolated from livers of Hmox1Alb-Cre and Hmox1fl/fl mice. The cells were cultured in serum-free media and treated with 38.44 μM heme arginate. The incubation was terminated at the indicated time intervals and the cells were harvested and used for RNA preparation. (A) qPCR analysis of Hmox1 mRNA. (B) qPCR analysis of Hamp mRNA. Data in graphs are presented as the mean ± SEM. Statistical analysis was performed by two-way ANOVA. Statistically significant differences across genotypes are indicated by *** (p<0.001). (TIF) [file pone.0219835.s001.tif]

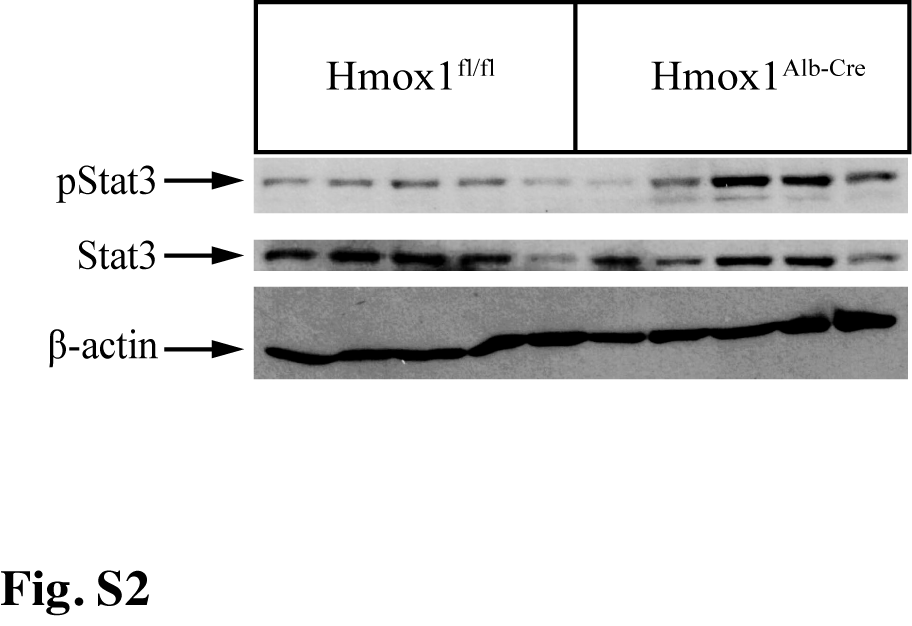

Supplement: S2 Fig — Primary hepatocytes were isolated from livers of Hmox1Alb-Cre and Hmox1fl/fl mice. The cells were cultured in serum-free media and then harvested and used for preparation of protein lysates. (A) Western blot analysis of pStat3, Stat3 and β-actin (arrows). (TIF) [file pone.0219835.s002.tif]
